# Uncited articles in Brazilian public health journals

**DOI:** 10.11606/S1518-8787.2017051000442

**Published:** 2017-11-16

**Authors:** Angela Maria Belloni Cuenca, Milena Maria de Araújo Lima Barbosa, Karoline de Oliveira, Fernanda Paranhos Quinta, Maria do Carmo Avamilano Alvarez, Ivan França

**Affiliations:** I Universidade de São Paulo. Faculdade de Saúde Pública. Departamento de Saúde, Ciclos de Vida e Sociedade. São Paulo, SP, Brasil; II Universidade de São Paulo. Faculdade de Saúde Pública. Programa de Pós-Graduação em Saúde Pública. São Paulo, SP, Brasil; III Universidade de São Paulo. Pró-Reitoria de Pesquisa. Bolsista do Programa Institucional de Bolsas de Iniciação Científica. São Paulo, SP, Brasil; IV Universidade de São Paulo. Faculdade de Saúde Pública. Biblioteca: Centro de Informação e Referência. São Paulo, SP, Brasil

**Keywords:** Journal Article, Scientific and Technical Publications, Citation Databases, Systems for Evaluation of Publications, Artigo de Revista, Publicações Científicas e Técnicas, Bases de Dados de Citações, Sistemas de Avaliação das Publicações

## Abstract

Here, we describe the percentage of non-citation in Brazilian public health journals, a field that, until now, had not been investigated nationally or internationally. We analyzed articles, published between 2008 and 2012, of eight public health journals indexed in the scopus database. The percentage of non-citation differs between journals (from 5.7% to 58.1%). We identified four statistically distinct groups: *História, Ciência, Saúde – Manguinhos* (58% uncited articles); *Physis: Revista de Saúde Coletiva, Interface,* and *Saúde e Sociedade* (32% to 37%); *Ciência & Saúde Coletiva* and *Revista Brasileira de Epidemiologia* (16% to 17%); and *Cadernos de Saúde Pública* and *Revista de Saúde Pública* (6%). The non-citation in the first three years post-publication also varies according to journal. Four journals have shown a clear decline of non-citation: *Cadernos de Saúde Pública, Ciência & Saúde Coletiva, Revista Brasileira de Epidemiologia*, and *Physis*. Another three (*Revista de Saúde Pública, Saúde e Sociedade*, and *Interface*) presented an oscillation in non-citation, but the rates of 2008 and 2012 are similar, with different magnitudes. In turn, the journal *História, Ciência, Saúde – Manguinhos* maintains high rates of non-citation. Multidisciplinary journals attract more citation, but a comprehensive citation model still needs to be formulated and tested.

## RESUMO

Aqui, descrevemos o percentual de não citação em revistas brasileiras de saúde pública, campo até agora não investigado nacional ou internacionalmente. Analisamos artigos, publicados entre 2008 e 2012, de oito revistas de saúde pública indexadas na base Scopus. O percentual de não citação difere entre as revistas (de 5,7% a 58,1%). Identificamos quatro grupos estatisticamente distintos: *História, Ciência, Saúde – Manguinhos* (58% de artigos não citados); *Physis: Revista de Saúde Coletiva, Interface*, e *Saúde e Sociedade* (32% a 37%); *Ciência & Saúde Coletiva* e *Revista Brasileira de Epidemiologia* (16% a 17%); e *Cadernos de Saúde Pública* e *Revista de Saúde Pública* (6%). A não citação nos primeiros três anos pós-publicação também varia segundo revista. Quatro revistas mostraram claro declínio da não citação: *Cadernos de Saúde Pública, Ciência & Saúde Coletiva, Revista Brasileira de Epidemiologia* e *Physis*. Outras três (*Revista de Saúde Pública, Saúde e Sociedade* e *Interface*) apresentaram oscilação na não citação, mas as taxas de 2008 e 2012 são similares, com magnitudes diferentes. Por sua vez, a revista *História, Ciência, Saúde – Manguinhos* mantém taxas elevadas de não citação. Revistas multidisciplinares atraem mais citação, mas um modelo compreensivo de citações ainda precisa ser formulado e testado.

## INTRODUCTION

From the beginning of the citation analysis, Garfield^1^ already showed concern about articles that were not cited. Hamilton^2^, in a citation analysis in the Web of Science, found 55% of uncited articles over the first five years post-publication. This proportion, however, varied according to the area of knowledge: 47.4% in the exact and biological sciences, 74.7% in the social sciences, and 98.0% in the arts and humanities^3^. A reanalysis of Hamilton’s data, restricted to original articles, estimated lower rates of non-citation: 22.4% (exact and biological sciences); 48% (social sciences); and 93% (arts and humanities)^4^. Although the non-citation rates estimated by Hamilton have been criticized^2^ ^-^ ^6^, the idea remains that, in fact, most of the articles were not cited or will not be cited^7^.

However, there are clear indications that non-citation has been continuously declining since the 1980s in the fields of natural sciences, engineering, medicine, and social sciences, but not in the humanities^7^.

The field of public health, multidisciplinary in nature, may be a relevant case for the evaluation of non-citation. However, it has not yet been investigated. Here, we describe the phenomenon of non-citation in Brazilian public health journals.

## METHODS

The selected journals had to obey two criteria: a) to be part of the Collective Health Editors Forum of the Brazilian Association of Collective Health (created in 2014, access at https://www.abrasco.org.br/site/2014/11/forum-de-editores-de-saude-coletiva-carta-de-sao-paulo/), which represents the Brazilian public health journals; and b) be indexed in the Scopus database, chosen by the multidisciplinary scope (more than 20 thousand journals indexed), relevance in the scientometric evaluation, and availability of the metadata for analysis. Thus, we selected eight journals (abbreviations by the National Library of Medicine Catalog - NLM): *Cadernos de Saúde Pública (*Cad Saude Publica*)*; *Ciência & Saúde Coletiva* (Cien Saude Colet); *História, Ciência, Saúde – Manguinhos* (Hist Cienc Saude); *Interface: Comunicação, Saúde, Educação* (Interface); *Physis: Revista de Saúde Coletiva* (Physis); *Revista Brasileira de Epidemiologia* (Rev Bras Epidemiol); *Revista de Saúde Pública* (Rev Saude Publica); and *Saúde e Sociedade* (Saude Soc).

From these, data were extracted (in November 2015) referring to two types of articles: the original articles and the reviews, published between 2008 and 2012. We chose 2008 as the initial date because all selected journals were indexed in Scopus. The end date (2015) ensured a minimum of three years for the most recent (published in 2012) to receive citations.

We retrieved 5,736 article records, of which 66 were deleted for duplicity, with a final sample of 5,670 articles. From each, the following information was obtained: article title, authorship, publication year, pagination, journal title, and number of citations of each article (per year and total).

To validate the consistency of the database, a subsample with an arbitrary value of 10% of the articles (567/5.670) was analyzed. We found some inconsistencies. The most frequent was the delay in indexing, i.e., new records of citations of articles appeared between the period of the sample extraction and that of the evaluation of the subsample. Thus, 60 articles presented late citations, equivalent to 10.6% of the 567. Of these 60 articles, four (0.7%) would no longer be classified as uncited. Other, less frequent, problems were: year of citation divergent from the date indicated in Scopus (0.5%) and a citation counted twice by Scopus (0.5%). The non-citation rate of the eight journals remained: 18.6% in the sample and 18.5% in the subsample. Given the small magnitude of problems and their insignificant effect on the estimates, the data studied were those extracted from the Scopus base, without corrections.

We calculated, for each article and in each journal, the proportion of non-citation in the whole period and the proportion of non-citation in the first three years after publication.

## RESULTS

**Figure 1:**
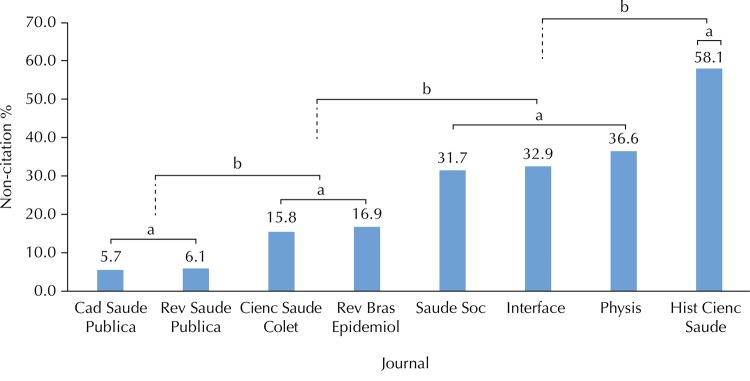
The proportion of uncited articles in Brazilian public health journals, published from 2008 to 2012. Scopus Database, 2015.

^a^ p > 0.05

^b^ p < 0.001

We observed a major difference in non-citation rates between the journals, ranging from 5.7% to 58.1%, a difference of 10 times between the lowest and the highest rates (Figure 1). Also noteworthy is the distribution in four statistically distinct groups: in the group with the highest non-citation, is the journal *Hist Cienc Saude*. Another group, comprised of social science health journals, included *Physis*, *Interface*, and *Saude Soc*. In an intermediate situation, we have *Cienc Saude Colet* and *Rev Bras Epidemiol*. The lowest non-citation indexes were identified in the *Cad Saude Publica* and *Rev Saude Publica* journals.

**Figure 2:**
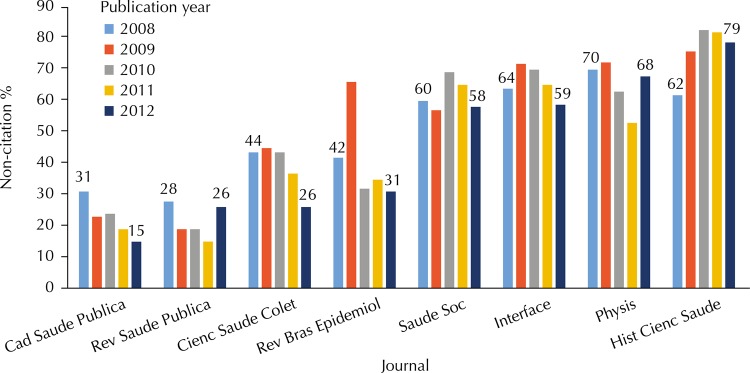
Evolution of non-citation rates in the three years following the publication of articles in Brazilian public health journals. Scopus Database, 2015.

The non-citation behavior in the first years after publication also varies according to the journal (Figure 2). It is interesting to note that the non-citation dynamics in the last three years differ from the groups observed in Figure 1. There are four journals with a clear decline in non-citation: *Cad Saude Publica*, *Cienc Saude Colet, Rev Bras Epidemiol,* and *Physis*. In other three (*Rev Saude Publica, Saude Soc,* and *Interface*), there is an oscillation in non-citation, but rates in 2012 are similar to those of 2008, albeit with different magnitudes. Finally, the journal *Hist Cienc Saude* maintains high rates of non-citation.

## DISCUSSION

Brazilian journals have marked differences between them in terms of non-citation but behave similarly to what has already been described in the literature regarding the peculiarities of each area. In fact, Pendlebury^4^, in an analysis of ISI-indexed journals between 1984 and 1988, already found these disparities between areas of knowledge, in which natural sciences, engineering, and medicine had lower rates, followed by the social sciences, and with the greatest non-citation in the humanities. Larivière et al.^7^ found similar groupings decades later.

The journal of humanities in the field of public health, which prioritizes the publication of articles related to documentation, research, and museum study in the history of science and health, has the highest overall non-citation rate but the rate has been decreasing in the first three years after publication. This pattern is comparable to the humanities journals analyzed in the Web of Science database^7^; that is, in the Brazilian case, 79% of articles not cited in *Hist Cienc Saude versus* 90% in the humanities journals mentioned by Larivière et al.^7^ Likewise, our health social science journals have similar behavior to those in the same field of knowledge indexed in the Web of Science, both in rates and in the reduction trend of non-citation.

On the other hand, it is noteworthy that our multidisciplinary public health journals (*Rev Saude Publica* and *Cad Saude Publica*) have comparatively lower levels of non-citation than those found by Larivière et al.^7^ These authors found a rate of uncited articles for medicine in general, and not their specialties, of around 11%. These differences can hardly be attributed to the different bibliometric sources, Scopus in our study and Web of Science in Larivière et al.^7^, because there is a high correlation between the impact indicators of these two bases^8^. The reasons why *Rev Saude Publica* and *Cad Saude Publica* are able to attract more citations still need to be studied, but they seem to follow the relationship in which the more interdisciplinary, the greater the ability to attract citations^9^.

Similarly, the journals *Cienc Saude Colet* and *Rev Bras Epidemiol*, both recent in the field of publishing in the area (created in 1996 and 1998, respectively), have similar characteristics regarding the overall rate of non-citation and the reduction of articles without citation in first three years. *Cienc Saude Colet* approaches multidisciplinary public health journals, with fewer articles not cited, probably due to its equally multidisciplinary nature. This journal undeniably has a strong tradition of disseminating production in the social sciences in health. *Rev Bras Epidemiol*, which publishes in an area that has the article as one of its main means of dissemination, has been attracting more and more citations. The monitoring of non-citations of these two journals, henceforth, may elucidate if they will be in the group of multidisciplinary journals.

Our analysis privileged non-citation, as we intend to bring up the proportion of articles that seem to have had no impact or recognition in the scientific community. Of course, this may be a limitation, since the analyses focused on the metrics of the citations (indicators such as index H, SJR, FI, cites per documents, among others) may reach different bibliometric classifications.

Pendlebury^4^ evaluates that a “certain level of non-citation in a journal is probably more an expression of the process of creation and dissemination of knowledge than any kind of performance measure,” recognizing that books are more important in the delivery of scientific communication among social and humanities scientists than in the exact and biological sciences. Thus, non-citation rates would represent more the forms of citing than the acceptance or rejection of the knowledge produced. The reasons for these differences have been speculated, but there are no comprehensive models to explain them. This gap opens room for further investigation.
